# Acute Effects of Monoacylglycerol Lipase Inhibitor ABX1431 on Neuronal Hyperexcitability, Nociception, Locomotion, and the Endocannabinoid System in HIV-1 Tat Male Mice

**DOI:** 10.1089/can.2023.0247

**Published:** 2024-12-02

**Authors:** Barkha J. Yadav-Samudrala, Havilah P. Ravula, Karenna M. Barmada, Hailey Dodson, Justin L. Poklis, Bogna M. Ignatowska-Jankowska, Aron H. Lichtman, Kathryn J. Reissner, Sylvia Fitting

**Affiliations:** ^1^ Department of Psychology and Neuroscience, University of North Carolina at Chapel Hill, Chapel Hill, North Carolina, USA.; ^2^ Department of Pharmacology and Toxicology, Virginia Commonwealth University, Richmond, Virginia, USA.; ^3^ Neuronal Rhythms in Movement Unit, Okinawa Institute of Science and Technology, Okinawa, Japan.

**Keywords:** monoacylglycerol lipase, neuroHIV, nociception, 2-arachidonoylglycerol, Tat transgenic mouse, arachidonic acid

## Abstract

**Background:**

Evidence suggests that monoacylglycerol lipase (MAGL) inhibitors can potentially treat HIV symptoms by increasing the concentration of 2-arachidonoylglycerol (2-AG). We examined a selective MAGL inhibitor ABX1431 in the context of neuroHIV.

**Methods:**

To assess the effects of ABX1431, we conducted *in vitro* and *in vivo* studies. *In vitro* calcium imaging on frontal cortex neuronal cultures was performed to evaluate the role of ABX1431 (10, 30, 100 nM) on transactivator of transcription (Tat)-induced neuronal hyperexcitability. Following *in vitro* experiments, *in vivo* experiments were performed using Tat transgenic male mice. Mice were treated with 4 mg/kg ABX1431 and assessed for antinociception using tail-flick and hot plate assays followed by locomotor activity. After the behavioral experiments, their brains were harvested to quantify endocannabinoids (eCB) and related lipids through mass spectrometry, and cannabinoid type-1 and -2 receptors (CB_1_R and CB_2_R) were quantified through western blot.

**Results:**

*In vitro* studies revealed that adding Tat directly to the neuronal cultures significantly increased intracellular calcium concentration, which ABX1431 completely reversed at all concentrations. Preincubating the cultures with CB_1_R and CB_2_R antagonists showed that ABX1431 exhibited its effects partially through CB_1_R. *In vivo* studies demonstrated that acute ABX1431 increased overall total distance traveled and speed of mice regardless of their genotype. Mass spectrometry and western blot analyses revealed differential effects on the eCB system based on Tat expression. The 2-AG levels were significantly upregulated following ABX1431 treatment in the striatum and spinal cord. Arachidonic acid (AA) was also upregulated in the striatum of vehicle-treated Tat(+) mice. No changes were noted in CB_1_R expression levels; however, CB_2_R levels were increased in ABX1431-treated Tat(−) mice only.

**Conclusion:**

Findings indicate that ABX1431 has potential neuroprotective effects *in vitro* partially mediated through CB_1_R. Acute treatment of ABX1431 *in vivo* shows antinociceptive effects, and seems to alter locomotor activity, with upregulating 2-AG levels in the striatum and spinal cord.

## Introduction

Approximately 39 million people were living with human immunodeficiency virus type-1 (HIV-1) globally, with 1.3 million newly infected in 2022.^1^ The advancement of combined antiretroviral therapies (cART) suppresses HIV-1 replication to undetectable levels^2^ but does not eradicate the virus entirely due to its low brain penetration.^3^ Low levels of viral replication and chronic immune activation still linger, leading to HIV-1-associated neurocognitive disorders (HAND).^4–6^ About 50% of cART-treated people living with HIV-1 (PLWH) develop some form of HAND, which includes problems with memory consolidation, attention,^7–9^ mood,^10,11^ reduced physical activity levels,^12^ and increased pain sensitivity.^13^ HAND is associated with synaptodendritic injury caused by inflammatory factors and viral proteins, including transactivator of transcription (Tat), released from infected and/or activated microglia/macrophages.^14^

The prevalence of HAND in the cART era raises questions about treating HIV-1-related brain disorders. The endocannabinoid (eCB) system presents promising therapeutic targets for neurocognitive disorders, such as HAND, due to its role in neuroprotection and anti-inflammatory processes.^15,16^ Endogenous cannabinoid ligands, *N*-arachidonoylethanolamine (anandamide; AEA) and 2-arachidonoylglycerol (2-AG), show neuroprotective effects in several preclinical models of neurodegenerative disease, including Parkinson's disease, Alzheimer's disease, and multiple sclerosis.^17–20^ In the context of neuroHIV, reviews have discussed the potential neuroprotective effects of eCB ligands.^21,22^ AEA and 2-AG mainly target cannabinoid type-1 and -2 receptors (CB_1_R and CB_2_R) and are rapidly degraded by fatty acid amide hydrolase (FAAH) and monoacylglycerol lipase (MAGL), respectively. As direct application of AEA and 2-AG have short durations of action,^23^ the strategy of inhibiting their hydrolytic enzymes possesses considerable promise.^24,25^

MAGL not only serves as the major 2-AG degradative enzyme but also is a major rate-limiting enzyme for producing arachidonic acid (AA).^26^ Accordingly, the MAGL inhibitor, JZL184, dampens inflammation in the central^27^ and peripheral^28^ nervous system by decreasing 2-AG metabolism and concomitantly reducing the production of its primary metabolite AA, as well as the further downstream proinflammatory prostaglandins.^29,30^ Moreover, JZL184 prevents gp-120-induced synapse loss through CB_2_R activation and downregulates production of inflammatory cytokines.^29^

The highly selective MAGL inhibitor, MJN110, displays neuroprotective effects *in vitro*, by reducing neuronal hyperexcitability and restoring dendritic arborization complexity, and *in vivo*, by mitigating Tat-induced neurocognitive alterations.^31^ These findings indicate that MAGL inhibitors represent a viable target for treating HAND. ABX1431 (renamed AG06466) is another highly selective MAGL inhibitor, which has undergone ∼16 clinical trials that have been terminated or completed^32,33^ and is considered safe to use.

ABX1431 significantly increases 2-AG concentration in the brain and produces antinociceptive effects in rats^34^ and also showed anticonvulsant effects in a mouse model of Dravet Syndrome by increasing hippocampal 2-AG concentration.^35^

Given that ABX1431 is a highly selective MAGL inhibitor with demonstrated safety in clinical trials, we examined its effects in the context of neuroHIV. Accordingly, the present study aims to determine the potential neuroprotective effects of ABX1431 against HIV Tat-induced neurotoxicity *in vitro* and its acute effects on various behavioral outcomes and the eCB system using the HIV Tat transgenic mouse model.

## Materials and Methods

Experiments and procedures described below were approved by the University of North Carolina at Chapel Hill and conducted following the National Institute of Health Guide and Use of Laboratory Animals.

### Primary neuronal culture and microglia conditioned media

**FIG. 1.**
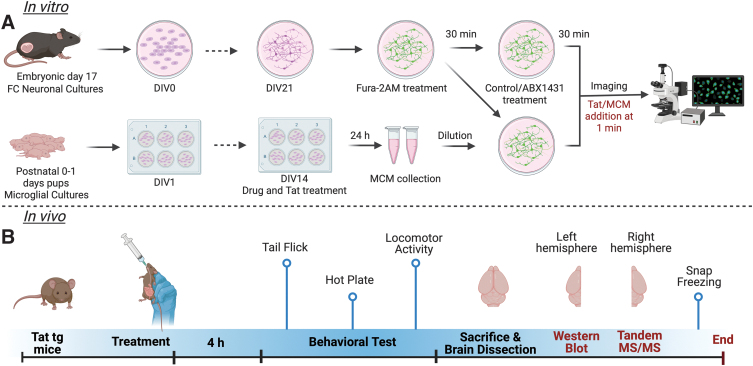
Overall outline of the study. **(A)** Steps involved in *in vitro* Ca^2+^ imaging studies using primary neuronal and microglial cultures. At DIV21 neurons were treated with Fura-2AM and the appropriate treatment condition, then incubated for 30 min and imaged. **(B)** *In vivo* study outline; Tat(−) and Tat(+) male mice were given ABX1431 (4 mg/kg) through oral gavage and after 4 h mice underwent the behavioral tests as shown. Ca^2+^, calcium; DIV, days *in vitro*; FC, frontal cortex; MCM, microglia conditioned media; Tat, transactivator of transcription; tg, transgenic. Figure created in Biorender.

Primary neuronal cultures (Fig. 1) were obtained from the frontal cortex (FC) of embryonic day 17 C57BL/6J mice (Charles River, Raleigh, NC) as previously described.^36,37^ Cultures were incubated in neurobasal media supplemented with appropriate nutrients at 37°C in a humidified atmosphere containing 5% CO_2_. All experiments were conducted at days *in vitro* (DIV) 21.

Primary microglial cultures were derived from whole brain of C57BL/6J (Charles River) pups at 0–1 postnatal days as described.^38^ After the treatments on DIV14, microglia conditioned media (MCM) were collected and frozen at −20°C. The MCM dilution used for the calcium (Ca^2+^) imaging experiments was 1:9.

### Live cell calcium imaging

Live fluorescence cell imaging was conducted on the cultured neurons using Zeiss Axio Observer Z.1 inverted microscope at 20×s objective (Carl Zeiss). Intracellular calcium ([Ca^2+^]_i_) was measured by treating neurons with a cell-permeant calcium indicator fura-2AM (k_d_=145 nM; 2.5 μM; Molecular Probes) as previously described.^37^ Neurons were incubated with appropriate treatments for 30 min prior imaging. Tat (100 nM) was bath applied to the cultures at the 1-, 3-, and 5-min mark and the excitation pattern was recorded for a total of 15 min. For the MCM experiments, 1 mL of diluted MCM was applied to the FC neuronal cultures at 1 min. The [Ca^2+^]_i_ production was calculated as previously described.^39^ At least three independent experiments were performed for each treatment. For quantitative analysis of ([Ca^2+^]_i_ levels, 12–15 neurons were randomly selected per treatment per experiment.

### Treatments

For *in vitro* experiments, primary FC neuronal and microglial cultures were treated with HIV-1 Tat_1–86_ (100 nM; ImmunoDiagnostic; clade 267B). Tat 100 nM concentration was used based on previous data from our laboratory.^31,37,38^ The concentrations of ABX1431 were based on previous activity-based protein profile studies.^34^ At DIV14 microglial cultures were incubated with ABX1431 for 1 h before Tat treatment. Treatment with CB_1_R antagonist rimonabant (100 nM; Tocris) and CB_2_R antagonist SR144528 (100 nM; Tocris) was done 30 min before the ABX1431 treatment. Tat was added to cultures and was allowed to incubate for 24 h before media collection.

For behavioral experiments ABX1431 (No. 26023; Cayman Chemical, Ann Arbor, MI) was dissolved in 1:1:18 mixture of ethanol:kolliphor:saline to obtain a 4 mg/kg solution. Based on previous studies,^34,40^ it has been determined that oral gavage of ABX1431 at 4 mg/kg significantly increased the 2-AG concentration in the mouse brain; therefore, ABX1431 dose of 4 mg/kg was selected for all the *in vivo* studies. Administration of vehicle or ABX1431 was done through oral gavage acutely 4 h before behavioral testing, and was randomized in all experiments.

### Animals

Doxycycline (DOX)-inducible, brain-restricted HIV-1_IIIB_ Tat_1–86_ transgenic male mice (∼7–9 months of age, *N*=28) were used and developed on a hybrid C57BL/6J background.^41,42^ Transgenic Tat(+) and control Tat(−) mice were fed a special DOX chow containing 6 mg/g DOX (Envigo) for 4 months before the experiment and were housed on a reversed 12-h light/12-h dark cycle.

### Behavioral procedure

All the behavioral experiments were blinded for treatment and genotype.

### Tail-flick and hot plate assay

ABX1431 4 mg/kg or vehicle were administered to the mice and spontaneous nociception was assessed 4 h posttreatment as previously described^43^ (Fig. 1). For tail-flick, the distal 1/3 of the tail was placed in a water bath maintained at 56°C±0.1°C. The latency to remove the tail from the bath was recorded. Hot plate was conducted immediately following the tail-flick assay. Mice were placed on the hot plate maintained at 55°C±0.1°C and removed immediately after withdrawing or licking a paw and the time was recorded. A maximum cutoff latency of 10 sec and 15 sec was used for tail-flick and hot plate, respectively, to prevent tissue damage.

### Locomotor activity

Mice were habituated to the locomotor activity chambers 24 h before the experiment and then placed into the experimental chambers as previously described^44^ (ENC-307W; 22×18 cm floor; MED Associates). Locomotion was recorded for 10 min using night vision cameras. Videos were analyzed using AnyMaze software.

### eCB and eicosanoid analysis

Following locomotor activity, animals were sacrificed by isoflurane-induced anesthesia followed by rapid decapitation. Prefrontal cortex (PFC), striatum, and spinal cord were dissected and snap frozen in liquid nitrogen. Endogenous cannabinoid ligands, including AEA, 2-AG, *N*-oleoylethanolamide (OEA), *N*-palmitoylethanolamide (PEA), and AA were quantified from samples of the right hemisphere using ultraperformance liquid chromatography–tandem mass spectrometry (UPLC-MS/MS) as previously described.^31,45^

### Western blot analysis

Western blot analysis was carried out on the same three central nervous system (CNS) regions from the left hemisphere as previously described.^46,47^ Briefly, tissue samples were homogenized on ice in Pierce™ RIPA lysis and extraction buffer (Thermo Scientific) with Halt™ protease and phosphatase inhibitor cocktail. BCA assay was performed on the lysates to determine the protein concentration. Proteins were denatured at 85°C for 10 min and equal amount of protein (20 μg/lane) was resolved at 120 volts for 1.5 h and transferred to nitrocellulose membrane at 1–4°C and 100 volts for 1 h. The membranes were incubated with Intercept blocking buffer at room temperature for 1 h followed by primary antibodies overnight at 4°C and secondary antibody for 1 h the next day.

The membranes were imaged and analyzed in Empiria studio software (LI-COR Biosciences). The following antibodies were used, primary antibodies: anti-CB_1_R (rabbit polyclonal; Proteintech), anti-CB_2_R (rabbit polyclonal; AbClonal), and anti-GAPDH antibody (mouse monoclonal; Abcam) as a housekeeping protein. Dilutions for primary are 1:1000, except GAPDH and secondary antibodies are 1:10,000 dilution.

Data represent the fold-change with respect to a control sample represented on all blots and normalized to the housekeeping gene GAPDH.

### Statistical analysis

All data are presented as mean±the standard error of the mean. *In vitro* data were analyzed using analyses of variance (ANOVAs). Animal data sets were analyzed by two-/three-way ANOVAs with time (1, 3, 8, 10 min) as a within-subjects factor and/or treatment (two levels: Vehicle, ABX1431) and genotype [two levels: Tat(−), Tat(+)] as between-subjects factors. All ANOVAs were followed up with Tukey's *post hoc* test when appropriate. An alpha level of *p*≤0.05 was considered significant for all statistical tests (Table 1). SPSS Statistics 28 and Prism GraphPad 8.0 were used for data analysis and data graphing, respectively.

**Table 1.** Statistical Data for All the Studies Conducted

**Study**	**Treatment effect, *p***	**Genotype effect, *p***	**Treatment×genotype effect, *p***	**Time effect, *p***	**Time×treatment effect, *p***	**Statistics**
*In vitro* studies						
Direct treatment to neurons	**<0.0001**	NA	NA	NA	NA	One-way ANOVA with Tukey's *post hoc* test
MCM treatment to neurons	**<0.0001**	NA	NA	NA	NA	
*In vivo* studies						
Hot plate	0.350	0.781	0.148	NA	NA	Two-way ANOVA with Tukey's *post hoc* test
Tail flick	**0.005**	0.870	**0.008**	NA	NA	
Total distance (m)	**0.041**	0.746	0.986	**<0.001**	**0.008**	Three-way ANOVA with Tukey's *post hoc* test
Average speed (m/sec)	**0.009**	0.704	0.942	**<0.001**	**0.047**	
Total time immobile (sec)	0.279	0.714	0.206	**0.001**	**<0.001**	
Mass spectrometry analysis						
PFC AEA	0.341	0.222	0.399	NA	NA	Two-way ANOVA with Tukey's *post hoc* test
PFC 2-AG	0.080	0.488	0.944	NA	NA	
PFC AA	0.194	0.562	0.818	NA	NA	
Str AEA	0.701	0.171	0.684	NA	NA	
Str 2-AG	**0.011**	0.270	0.778	NA	NA	
Str AA	0.299	**0.005**	**0.053**	NA	NA	
SC AEA	0.791	0.304	0.808	NA	NA	
SC 2-AG	**0.013**	0.907	0.984	NA	NA	
SC AA	0.987	0.754	0.334	NA	NA	
Western blot analysis						
PFC CB_1_R	0.086	0.339	0.304	NA	NA	Two-way ANOVA with Tukey's *post hoc* test
PFC CB_2_R	0.119	0.112	0.104	NA	NA	
Str CB_1_R	0.188	0.295	0.117	NA	NA	
Str CB_2_R	**0.001**	0.109	**0.035**	NA	NA	
SC CB_1_R	0.095	0.444	0.332	NA	NA	
SC CB_2_R	0.055	0.134	0.115	NA	NA	

Bolded values denote significance at *p*≤0.05.

2-AG, 2-arachidonoylglycerol; AA, arachidonic acid; AEA, *N*-arachidonoylethanolamine; ANOVA, analysis of variance; CB_1_R, cannabinoid type-1 receptor; CB_2_R, cannabinoid type-2 receptor; MCM, microglia conditioned media; NA, not applicable; PFC, prefrontal cortex; SC, spinal cord; Str, striatum.

## Results

### *In vitro* studies

**FIG. 2.**
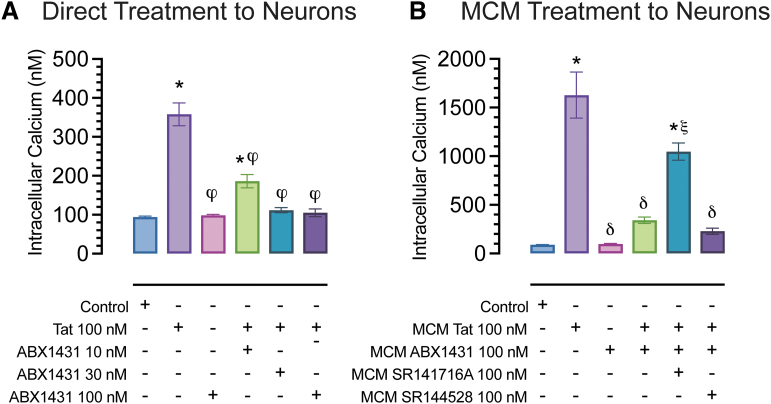
Calcium changes in cultured FC neurons at DIV21. **(A)** Direct Tat and drug treatment onto neuronal cultures. The graph represents changes in [Ca^2+^]_i_ spikes upon 100 nM Tat administration. The significant increase in [Ca^2+^]_i_ levels by Tat was significantly decreased by ABX1431 pretreatments (10, 30, 100 nM) in a concentration-dependent manner. **(B)** Neuronal cultures exposed to MCM derived from Tat and drug-treated microglia. The graph represents changes in [Ca^2+^]_i_ levels of neuronal cultures upon MCM treatment. MCM Tat 100 nM caused a significant increase in [Ca^2+^]_i_ levels, while MCM ABX1431+Tat did not, indicating that ABX1431 100 nM blocked the effects of MCM Tat. Additionally, CB_1_R or CB_2_R antagonists, rimonabant and SR144528, respectively, did not block ABX1431's protective effects as a significant downregulation in [Ca^2+^]_i_ levels was noted for both conditions compared with MCM Tat. However, CB_1_R seems to partially be involved in ABX1431's protective effects as MCM Tat+ABX1431+rimonabant significantly differed from control. All data are expressed as mean ± the SEM. Statistical significance was assessed by ANOVAs followed by Tukey's *post hoc* tests; *p<0.0001 versus control, ^ϕ^*p*<0.0001 versus Tat, ^δ^*p*<0.0001 and ^ξ^*p*=0.001 versus MCM Tat. ANOVA, analysis of variance; [Ca^2+^]_i_, intracellular calcium; CB_1_R, cannabinoid type-1 receptor; CB_2_R, cannabinoid type-2 receptor; FC, frontal cortex.

To study the role of ABX1431 in the context of neuroHIV, we conducted *in vitro* Ca^2+^ imaging studies using primary FC neuronal cultures at DIV21. Tat (100 nM) application onto neuronal cultures significantly increased [Ca^2+^]_i_ concentration, which was significantly reduced by ABX1431 pretreatment at all concentrations (10, 30, 100 nM; *p*<0.0001; Fig. 2A). Similarly, to understand the role of MAGL inhibition and Tat in mediating microglial neurotoxicity, we assessed [Ca^2+^]_i_ responses of FC neuronal cultures to MCM derived from ABX1431 and/or Tat-treated microglia in the presence and absence of CB_1_R (rimonabant) and CB_2_R (SR144528) antagonists.

Application of MCM Tat onto neurons resulted in a significant increase in [Ca^2+^]_i_^,^ (*p*<0.001), which was blocked completely when applying MCM cotreated with ABX1431 and Tat (*p*<0.0001). MCM derived from cotreatment of ABX1431, Tat, and rimonabant only showed partial neuroprotective effects. Whereas MCM derived from cotreatment of ABX1431, Tat, and SR144528 showed protective effects similar to that of MCM derived from Tat and ABX1431 treatment, which suggests the protective effects of ABX1431 are partially CB_1_R mediated only and not by CB_2_R (Fig. 2B).

### *In vivo* studies

**FIG. 3.**
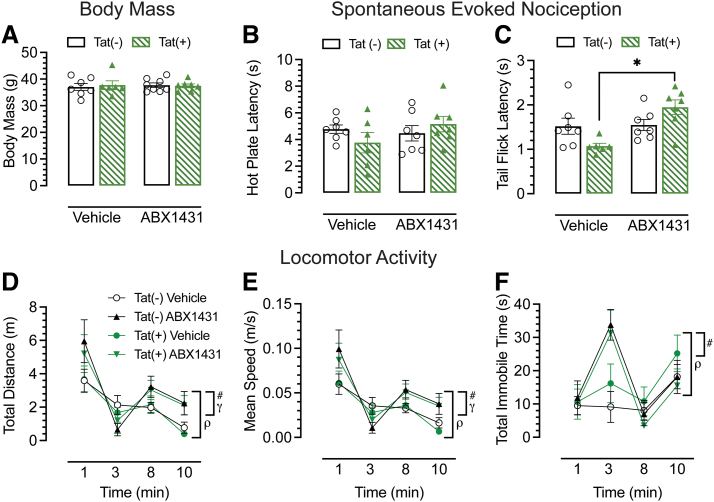
Effect of acute ABX1431 at 4 mg/kg on body mass and spontaneous evoked nociception and locomotor activity. **(A)** Body mass was not affected by acute ABX1431 dose. **(B)** For the hot plate assay, no effects were noted by acute ABX1431 administration. **(C)** For the spinal-related tail-flick assay, ABX1431 administration at 4 mg/kg increased latency to pain signals in Tat(+) mice, whereas no significant differences were found in Tat(−) mice. **(D)** Locomotor activity was significantly altered by acute ABX1431 administration over 10 min and increased overall locomotor activity. **(E)** ABX1431 significantly changed the average speed by which mice traveled and increased overall speed of mice. **(F)** ABX1431 caused increase in immobility especially at 3-min time point irrespective of the genotype. All data are expressed as mean±the SEM. Statistical significance was assessed by ANOVAs followed by Tukey's *post hoc* tests; *p=0.002 vehicle-treated Tat(+) mice versus ABX1431-treated Tat(+) mice for tail-flick assay; ^#^*p*≤0.001 main time effect; ^γ^*p*<0.05 main treatment effect; ^ρ^*p*<0.05 time×treatment interaction effect.

Tat(−) and Tat(+) male mice were randomly assigned to vehicle or ABX1431-treated groups. No differences were observed in body mass between groups (Fig. 3A). The hot plate and tail-flick assays were conducted 4 h after ABX1431 oral gavage (4 mg/kg) to access heat-evoked nociception. Hot plate assesses supraspinal-related nociception, and no drug or genotype-dependent effects were observed (Fig. 3B). For tail-flick, spinal-related nociception, acute ABX1431 significantly increased pain latencies in Tat(+) male mice only (*p*=0.002; Fig. 3C).

Furthermore, to evaluate effects of acute ABX1431 (4 mg/kg) on motor function, we assessed locomotor activity (Fig. 3D) following the hot plate assay. Over the period of 10 min, there was a significant decrease in locomotion (*p*<0.001); however, when treatment effect was considered, ABX1431 significantly altered this decrease in locomotor activity over the 10 min (*p*=0.008). Interestingly, ABX1431 increased the overall locomotor activity (*p*=0.041). Similarly, there were significant differences in average speed (Fig. 3E), with a significant decrease in speed over time (*p*<0.001), which was altered by acute ABX1431 (*p*=0.047). Furthermore, acute ABX1431 exposure significantly increased overall speed (*p*=0.009). Finally, there were no changes in the overall immobility due to ABX1431 (Fig. 3F), but increased immobility over time (*p*<0.001) was significantly altered by ABX1431 (*p*<0.001), which was specifically noted for the 3-min time point, where ABX1431 increased the immobility in both the genotypes.

### CNS levels of eCB and receptors

**FIG. 4.**
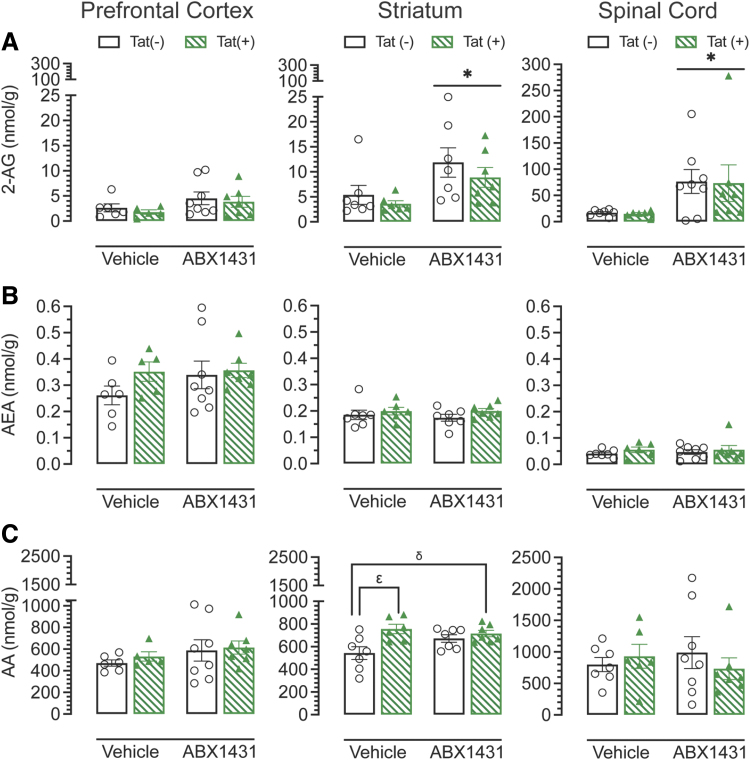
Endocannabinoid levels (nmol/g) in prefrontal cortex, striatum, and spinal cord. **(A)** 2-AG levels were found to be lowest in the prefrontal cortex and ABX1431 caused a significant upregulation in 2-AG levels in both, striatum and spinal cord, for both genotypes. **(B)** Acute ABX1431 treatment did not alter AEA levels in any regions regardless of genotype and drug treatment. **(C)** AA levels were comparable between the three brain regions and only striatum showed a significant drug×genotype interaction with vehicle-treated Tat(+) mice showing a significant upregulation in AA levels as compared with vehicle-treated Tat(−) mice and no change was observed with ABX1431 treatment. All data are expressed as mean±the SEM. Statistical significance was assessed by ANOVAs followed by Tukey's *post hoc* test where appropriate; *p≤0.05 main effect of drug; ^δ^*p*=0.031 genotype×drug effect; ^ɛ^*p*=0.009 vehicle-treated Tat(−) mice versus vehicle-treated Tat(+) mice. 2-AG, 2-arachidonoylglycerol; AA, arachidonic acid; AEA, *N*-arachidonoylethanolamine.

Following behavioral experiments, CNS regions (PFC, striatum, spinal cord) were taken, quantified, and analyzed by two-way ANOVA for eCB and eicosanoid levels through UPLC-MS/MS, including AEA, 2-AG, PEA, OEA, and AA (Fig. 4 and Supplemental Table S1). Lipid concentrations (nmol/g) significantly varied between CNS regions (Fig. 4). The order of 2-AG levels was PFC < striatum < spinal cord. Importantly, acute ABX1431 significantly upregulated 2-AG levels in the striatum and spinal cord regardless of genotype (*p*<0.05). For AEA, no significant drug or genotype effect was noted with the order of AEA levels being PFC > striatum > spinal cord. Finally, AA levels were comparable between CNS regions, with only the striatum showing a drug×genotype interaction (*p*=0.031). AA was significantly upregulated in vehicle-treated Tat(+) males compared with Tat(−) males (*p*=0.009).

**FIG. 5.**
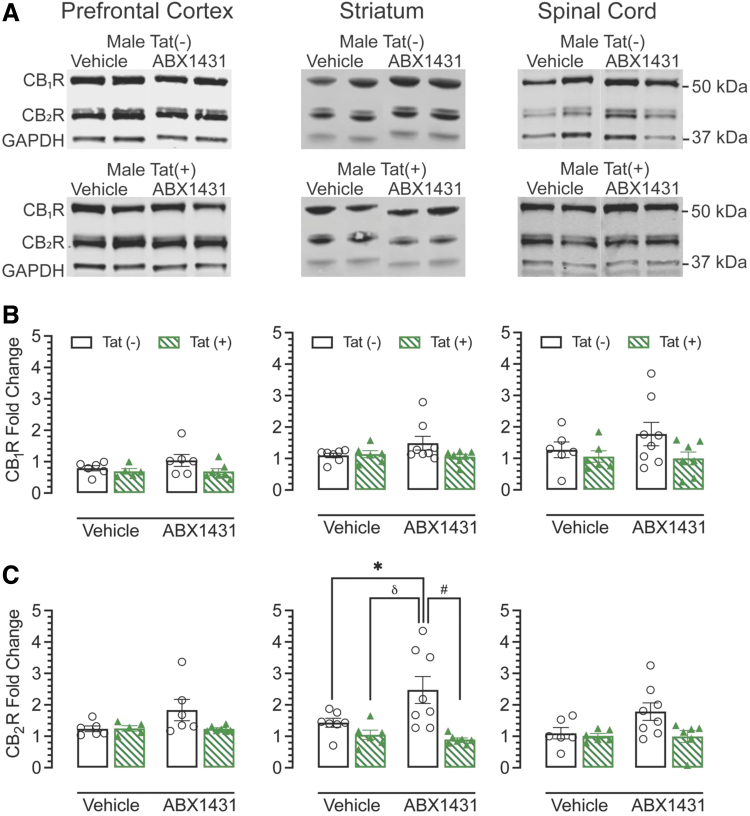
Cannabinoid receptors 1 and 2 expression levels in prefrontal cortex, striatum, and spinal cord. CB_1_R and CB_2_R expression levels were assessed in PFC, striatum, and spinal cord of vehicle and acute ABX1431-treated Tat transgenic mice through western blot. **(A)** Representative immunoblots for CB_1_R, CB_2_R, and housekeeping protein GAPDH. **(B)** No genotype-specific and treatment-specific alterations were found in CB_1_R levels in any CNS regions. **(C)** Significant changes in CB_2_R levels were found in the striatum. ABX1431 treatment significantly increased CB_2_R levels in Tat(−) mice and no changes were observed in Tat(+) mice. All data are expressed as mean±the SEM. Statistical significance was assessed by ANOVAs followed by Tukey's *post hoc* test where appropriate; *p=0.004 main drug effect; ^#^*p*=0.001 main genotype effect; ^δ^*p*=0.005 genotype×drug effect. CNS, central nervous system.

Regions from the left hemisphere were used for western blot analysis (Fig. 5). No alterations were found for CB_1_R protein expression levels (Fig. 5B). A significant treatment×genotype effect was seen for CB_2_R expression levels in the striatum (Fig. 5C).

CB_2_R was upregulated in ABX1431-treated Tat(−) mice (*p*=0.004) and no changes were seen in Tat(+) mice. Raw unedited western blot images have been added to Supplemental Figure S1.

## Discussion

HIV-1 Tat activates the glutamatergic *N*-methyl-*D*-aspartate (NMDA) receptors^36,48–50^ and interacts with the lipoprotein receptor-related protein,^51^ causing an increase in neuronal intracellular calcium, dendritic damage, and synapse loss.^49,52–54^ 2-AG and AEA have been previously reported to counter these effects of Tat by downregulating intracellular calcium concentrations.^47^ In contrast to AEA, 2-AG is present at higher concentrations in the brain and acts as a full agonist at both CB_1_R and CB_2_R.^55–58^ The 2-AG is primarily hydrolyzed by MAGL into AA and glycerol, the primary source for cyclooxygenase-2 mediated production of proinflammatory prostaglandins.^26,59^ Therefore, the neuroprotective effects of MAGL inhibitors may be due to reduced neuroinflammatory responses because of decreased production of AA and its prostaglandin metabolites, while simultaneously elevating 2-AG to enhance eCB receptor signaling.^59^

Most *in vivo* studies have used shorter Tat exposure times of ≤2 weeks, but here we chose 16-week exposure to model the chronic elevation reflected human disease. While some studies have reported Tat-induced allodynia, hyperalgesia and damaged nerve fibers,^60–62^ other studies have found either no effects^63^ or even decreased pain sensitivity.^13^ The effect of Tat on nociception is complex and not clearly understood.^13^ Pain sensitivity also depends on the length of Tat exposure, with studies showing hyposensitivity following 3 weeks of Tat induction^13^ and hypersensitivity after over a month of Tat exposure.^60,64^

The hyposensitivity may be due to neuronal dysfunction with initial Tat exposure, which is later reversed into hypersensitivity after prolonged exposure.^43^ It is plausible that von Frey filaments (i.e., mechanical allodynia) may be a more sensitive assay to detect Tat-induced hypernociception compared with the hot plate or tail-flick assays. Various MAGL inhibitors have been shown to attenuate pain primarily through CB_1_R activation.^65–69^ The present study also showed that an acute ABX1431 (4 mg/kg) dose increased pain latency in Tat(+) male mice.

The mechanisms by which MAGL inhibitors exert their antinociceptive effects include the involvement of CB_1_R^67,70,71^ or CB_2_R^72^ while others suggest the involvement of both CB_1_R and CB_2_R.^71,73,74^ Moreover, another study claims that the therapeutic effects of MAGL inhibitor JZL184 is anti-inflammatory and does not depend on cannabinoid receptors.^75^ We did not see any alterations in CB_1_R levels, although 2-AG was upregulated in the striatum and spinal cord.

There is a possibility that the CB_1_R functional activity may be altered, which cannot be detected by western blot. On the contrary, it is also possible that acute drug treatment was insufficient for substantial 2-AG-induced CB_1_R receptor desensitization and subsequent internalization or loss of surface receptors as loss of CB_1_R is demonstrated after prolonged use or chronic exposure to MAGL inhibitors.^76^ These differences in the mechanism of action of MAGL inhibitors could be explained by the transient role of CB_1_Rs resulting from 2-AG overload leading to desensitization of cannabinoid receptors following excessive MAGL inhibition.^76,77^

Motor deficits, including impaired gait, motor strength, and motor coordination, are comorbidities PLWH face^78,79^ leading to physical ailments and inadequate daily activity levels, even lower than most other chronic diseases.^12^ Preclinical studies have revealed that the presence of Tat significantly decreases locomotion,^80–83^ which is believed to be due to significant changes in the synaptic organization after Tat exposure.^80^ However, we did not find any genotype differences in locomotor activity and speed in our vehicle-treated mice, which is in line with some past studies.^84,85^

Discrepancies between studies may arise due to shorter open-field experiment duration, which was 10 min in our study and 30 min in a previously reported study,^80^ or due to longer Tat exposure (6 months).^81^ Perhaps studies such as grip strength may have detected genotype differences. For ABX1431, we found that acute ABX1431 significantly increased overall locomotion and speed of mice regardless of their genotype, which supports previous studies where treatment with MAGL inhibitors, including JZL184 and MJN110, increased locomotor activity.^86–88^

Differential effects of JZL184 and MJN110 have also been reported, in which JZL184 decreased locomotion, whereas MJN110 increased speed and total distance traveled.^71^ These differences in locomotor activity in the presence of various MAGL inhibitors and doses are yet to be understood and require further investigation.

Changes in the eCB system in PLWH have been previously reported.^89,90^ There is little information about the levels of endogenous ligands such as AEA and 2-AG in neuroHIV. Although we did not see any changes in AEA levels in the CNS regions, ABX1431 significantly increased 2-AG in striatum and spinal cord of both genotypes. The increase in 2-AG levels in the spinal cord may account for the increased latency in the tail-flick assay in ABX1431-treated mice.

Additionally, AA, known to be a key player in inflammatory responses,^91–93^ was significantly upregulated in vehicle-treated Tat(+) mice, suggesting an increase in proinflammatory mediators in the presence of Tat.^94^ This is supported by a recent study where AA cascade and eicosanoid production were upregulated in the brains of HIV-1 gp120 mice.^95^ As ABX1431 is a MAGL inhibitor, we showed 2-AG levels to be upregulated, but AA levels did not change. The modest increase of 2-AG and lack of changes in AA may be due to the route of administration of ABX1431, as previous studies have shown that intraperitoneal injection of JZL184 caused a large increase in 2-AG and decrease in AA concentration.^96^

Also, the dose of JZL184 used in the previous study^96^ (40 mg/kg) was much higher than the present study, which may be responsible for a decrease in AA concentration. It is also important to note that although MAGL is the primary enzyme responsible for the metabolism of 2-AG, serine hydrolases, α/β hydrolase domain 6 and 12 (ABHD6 and 12) are two other enzymes that play a minor but significant role in 2-AG hydrolysis,^97,98^ which may be responsible for the increase in AA levels. It is also important to consider that lack of changes in AA may be due to the Tat-induced proinflammatory effect, which releases AA from cell membrane phospholipids through mechanism such as tumor necrosis factor and toll-like receptors, which are independent from MAGL.^99^ Finally, we did not see any genotype-based changes in the CB_1_R and CB_2_R levels, except for CB_2_R expression in the striatum, which was downregulated in vehicle-treated Tat(+) mice when compared with Tat(−) mice.

The lack of CB_1_R alterations in neuroHIV has been reported previously^90,100,101^; however, most studies^89,100,102^ have shown an upregulation in CB_2_R in the context of neuroHIV due to its inducible nature upon microglial cell activation leading to its anti-inflammatory function.^103–105^ Interestingly, we found that acute ABX1431 administration led to upregulated CB_2_R expression in the striatum of Tat(−) mice without affecting Tat(+) mice, which is the opposite of previous research findings where desensitization of the GPCRs was caused by 2-AG overload.^43,76,77^ The exact relationship between the effects of MAGL inhibitors on the eCB system and its signaling pathway is yet to be understood, and warrants further investigation.

This study has several limitations; first, we only used male mice in the study. Literature has shown that women are more vulnerable to HAND symptoms.^106–111^ Therefore, it is critical to understand the role of MAGL inhibitors using female mice along with the consideration of estrous cycle in future investigations. Second, the HIV Tat transgenic model used in this study is a well-established neuroHIV model; however, it only expresses one of many viral proteins in HIV. Therefore, this is an important consideration when generalizing findings to PLWH, as viral proteins may interact, target various signaling pathways, and modify the CNS compared with a single protein.^94^

Lastly, the use of DOX to induce Tat expression in the Tat transgenic mouse is another limiting factor as DOX on its own has shown to exert neuroprotective effects and might mask some of the effects seen in this study.^112,113^ To minimize bias and confound, all animals, including the Tat(−) mice were fed the same DOX chow throughout the study. Furthermore, it would be important to investigate the chronic effects of ABX1431 exerted on nociception, locomotor activity, and eCB levels, along with other comorbidities of HAND, such as anxiety and memory.

## Conclusion

In conclusion, the present study demonstrates *in vitro* neuroprotective effects of a potent MAGL inhibitor on selected behaviors in a neuroHIV mouse model. Specifically, ABX1431 downregulated Tat- and MCM Tat-induced intracellular calcium release through a CB_1_R-mediated mechanism. Moreover, acute ABX1431 displayed moderate antinociceptive effects and increased locomotor activity in our HIV Tat transgenic mouse model. While ABX1431 caused an increase in 2-AG levels in the striatum and spinal cord, only CB_2_R was found to be upregulated in the striatum. Mechanistic studies are required to investigate how these alterations in the eCB system by MAGL inhibitors correlate with the behavioral outcomes in the context of neuroHIV. Based on previous clinical studies, ABX1431 is safe to use and therefore repurposing it for the treatment of HAND would facilitate future clinical trials.

## Abbreviations Used

2-AG: 2-arachidonoylglycerol
AA: arachidonic acid
AEA: *N*-arachidonoylethanolamine
ANOVA: analysis of variance
Ca^2+^: calcium
[Ca^2+^]_i_: intracellular calcium
cART: combined antiretroviral therapy
CB_1_R: cannabinoid type-1 receptor
CB_2_R: cannabinoid type-2 receptor
CNS: central nervous system
DIV: days *in vitro*
DOX: doxycycline
eCB: endocannabinoid
FC: frontal cortex
HAND: HIV-1-associated neurocognitive disorders
HIV-1: human immunodeficiency virus type-1
JSPS: Japan Society for Promotion of Science
MAGL: monoacylglycerol lipase
MCM: microglia conditioned media
NA: not applicable
OEA: *N*-oleoylethanolamide
PEA: *N*-palmitoylethanolamide
PFC: prefrontal cortex
PLWH: people living with HIV-1
SC: spinal cord
SEM: standard error of the mean
Str: striatum
Tat: transactivator of transcription
tg: transgenic
UPLC-MS/MS: ultraperformance liquid chromatography–tandem mass spectrometry

## References

[1] Joint United Nations Programme on HIV/AIDS (UNAIDS). Global HIV & AIDS Statistics—Fact Sheet. Geneva, Switzerland; 2023. Available from: https://home.liebertpub.com/publications/cannabis-and-cannabinoid-research/633/for-authors [Last accessed: December 24, 2023].

[2] Peltenburg NC, Schoeman JC, Hou J, et al. Persistent metabolic changes in HIV-infected patients during the first year of combination antiretroviral therapy. Sci Rep 2018;8(1):16947; doi: 10.1038/s41598-018-35271-0 30446683 PMC6240055

[3] Martinez-Picado J, Deeks SG. Persistent HIV-1 replication during antiretroviral therapy. Curr Opin HIV AIDS 2016;11(4):417–423; doi: 10.1097/COH.0000000000000287 27078619 PMC4900428

[4] Gannon P, Khan MZ, Kolson DL. Current understanding of HIV-associated neurocognitive disorders pathogenesis. Curr Opin Neurol 2011;24(3):275–283; doi: 10.1097/WCO.0b013e32834695fb 21467932 PMC3683661

[5] Heaton RK, Franklin DR, Ellis RJ, et al. HIV-associated neurocognitive disorders before and during the era of combination antiretroviral therapy: Differences in rates, nature, and predictors. J Neurovirol 2011;17(1):3–16; doi: 10.1007/s13365-010-0006-1 21174240 PMC3032197

[6] Sacktor N, McDermott MP, Marder K, et al. HIV-associated cognitive impairment before and after the advent of combination therapy. J Neurovirol 2002;8(2):136–142; doi: 10.1080/13550280290049615 11935465

[7] Cysique LA, Maruff P, Brew BJ. Prevalence and pattern of neuropsychological impairment in human immunodeficiency virus-infected/acquired immunodeficiency syndrome (HIV/AIDS) patients across pre- and post-highly active antiretroviral therapy eras: A combined study of two cohorts. J Neurovirol 2004;10(6):350–357; doi: 10.1080/13550280490521078 15765806

[8] Gartner S. HIV infection and dementia. Science 2000;287(5453):602–604; doi: 10.1126/science.287.5453.602 10691542

[9] Scott JC, Woods SP, Carey CL, et al. Neurocognitive consequences of HIV infection in older adults: An evaluation of the “cortical” hypothesis. AIDS Behav 2011;15(6):1187–1196; doi: 10.1007/s10461-010-9815-8 20865313 PMC3110599

[10] Bing EG, Burnam MA, Longshore D, et al. Psychiatric disorders and drug use among human immunodeficiency virus-infected adults in the United States. Arch Gen Psychiatry 2001;58(8):721–728; doi: 10.1001/archpsyc.58.8.721 11483137

[11] Orlando M, Burnam MA, Beckman R, et al. Re-estimating the prevalence of psychiatric disorders in a nationally representative sample of persons receiving care for HIV: Results from the HIV Cost and Services Utilization Study. Int J Methods Psychiatr Res 2002;11(2):75–82; doi: 10.1002/mpr.125 12459797 PMC6878230

[12] Vancampfort D, Mugisha J, De Hert M, et al. Global physical activity levels among people living with HIV: A systematic review and meta-analysis. Disabil Rehabil 2018;40(4):388–397; doi: 10.1080/09638288.2016.1260645 27929355

[13] Bagdas D, Paris JJ, Carper M, et al. Conditional expression of HIV-1 tat in the mouse alters the onset and progression of tonic, inflammatory and neuropathic hypersensitivity in a sex-dependent manner. Eur J Pain 2020;24(8):1609–1623; doi: 10.1002/ejp.1618 32533878 PMC7856573

[14] Kovalevich J, Langford D. Neuronal toxicity in HIV CNS disease. Future Virol 2012;7(7):687–698; doi: 10.2217/fvl.12.57 23616788 PMC3632417

[15] Compagnucci C, Di Siena S, Bustamante MB, et al. Type-1 (CB1) cannabinoid receptor promotes neuronal differentiation and maturation of neural stem cells. PLoS One 2013;8(1):e54271; doi: 10.1371/journal.pone.0054271 23372698 PMC3553153

[16] Howlett AC. Cannabinoid receptor signaling. Handb Exp Pharmacol 2005(168):53–79; doi: 10.1007/3-540-26573-2_2 16596771

[17] Tanveer R, McGuinness N, Daniel S, et al. Cannabinoid receptors and neurodegenerative diseases. WIREs MembrTransp Signal 2012;1:633–639.

[18] Bhunia S, Kolishetti N, Arias AY, et al. Cannabidiol for neurodegenerative disorders: A comprehensive review. Front Pharmacol 2022;13:989717; doi: 10.3389/fphar.2022.989717 36386183 PMC9640911

[19] Scotter EL, Abood ME, Glass M. The endocannabinoid system as a target for the treatment of neurodegenerative disease. Br J Pharmacol 2010;160(3):480–498; doi: 10.1111/j.1476-5381.2010.00735.x 20590559 PMC2931550

[20] Pertwee RG. Elevating endocannabinoid levels: Pharmacological strategies and potential therapeutic applications. Proc Nutr Soc 2014;73(1):96–105; doi: 10.1017/S0029665113003649 24135210

[21] Wu MM, Zhang X, Asher MJ, et al. Druggable targets of the endocannabinoid system: Implications for the treatment of HIV-associated neurocognitive disorder. Brain Res 2019;1724:146467; doi: 10.1016/j.brainres.2019.146467 31539547 PMC6880862

[22] Yadav-Samudrala BJ, Fitting S. Mini-review: The therapeutic role of cannabinoids in neuroHIV. Neurosci Lett 2021;750:135717; doi: 10.1016/j.neulet.2021.135717 33587986 PMC7994193

[23] Maccarrone M, Finazzi-Agro A. The endocannabinoid system, anandamide and the regulation of mammalian cell apoptosis. Cell Death Differ 2003;10(9):946–955; doi: 10.1038/sj.cdd.4401284 12934069

[24] Ahn K, McKinney MK, Cravatt BF. Enzymatic pathways that regulate endocannabinoid signaling in the nervous system. Chem Rev 2008;108(5):1687–1707; doi: 10.1021/cr0782067 18429637 PMC3150828

[25] Lichtman AH, Blankman JL, Cravatt BF. Endocannabinoid overload. Mol Pharmacol 2010;78(6):993–995; doi: 10.1124/mol.110.069427 20952498 PMC2993463

[26] Nomura DK, Morrison BE, Blankman JL, et al. Endocannabinoid hydrolysis generates brain prostaglandins that promote neuroinflammation. Science 2011;334(6057):809–813; doi: 10.1126/science.1209200 22021672 PMC3249428

[27] Rahmani MR, Shamsizadeh A, Moghadam-Ahmadi A, et al. JZL184, as a monoacylglycerol lipase inhibitor, down-regulates inflammation in a cannabinoid pathway dependent manner. Biomed Pharmacother 2018;103:1720–1726; doi: 10.1016/j.biopha.2018.05.001 29864962

[28] Sakin YS, Dogrul A, Ilkaya F, et al. The effect of FAAH, MAGL, and dual FAAH/MAGL inhibition on inflammatory and colorectal distension-induced visceral pain models in rodents. Neurogastroenterol Motil 2015;27(7):936–944; doi: 10.1111/nmo.12563 25869205

[29] Zhang X, Thayer SA. Monoacylglycerol lipase inhibitor JZL184 prevents HIV-1 gp120-induced synapse loss by altering endocannabinoid signaling. Neuropharmacology 2018;128:269–281; doi: 10.1016/j.neuropharm.2017.10.023 29061509 PMC5752128

[30] Muldoon PP, Akinola LS, Schlosburg JE, et al. Inhibition of monoacylglycerol lipase reduces nicotine reward in the conditioned place preference test in male mice. Neuropharmacology 2020;176:108170; doi: 10.1016/j.neuropharm.2020.108170 32479813 PMC7529882

[31] League AF, Gorman BL, Hermes DJ, et al. Monoacylglycerol lipase inhibitor MJN110 reduces neuronal hyperexcitability, restores dendritic arborization complexity, and regulates reward-related behavior in presence of HIV-1 Tat. Front Neurol 2021;12:651272; doi: 10.3389/fneur.2021.651272 34484091 PMC8415271

[32] NIH National Library of Medicine. Clinical studies for Lu AG06466. Bethesda, MD. Available from: https://clinicaltrials.gov/search?cond=AG06466&limit=50 [Last accessed: October 23, 2023].

[33] NIH National Library of Medicine. Clinical studies for ABX-1431. Bethesda, MD. Available from: https://clinicaltrials.gov/search?cond=abx1431&limit=50&page=1 [Last accessed: October 23, 2023].

[34] Cisar JS, Weber OD, Clapper JR, et al. Identification of ABX-1431, a selective inhibitor of monoacylglycerol lipase and clinical candidate for treatment of neurological disorders. J Med Chem 2018;61(20):9062–9084; doi: 10.1021/acs.jmedchem.8b00951 30067909

[35] Anderson LL, Doohan PT, Hawkins NA, et al. The endocannabinoid system impacts seizures in a mouse model of Dravet syndrome. Neuropharmacology 2022;205:108897; doi: 10.1016/j.neuropharm.2021.108897 34822817 PMC9514665

[36] Fitting S, Knapp PE, Zou S, et al. Interactive HIV-1 Tat and morphine-induced synaptodendritic injury is triggered through focal disruptions in Na(+) influx, mitochondrial instability, and Ca(2)(+) overload. J Neurosci 2014;34(38):12850–12864; doi: 10.1523/JNEUROSCI.5351-13.2014 25232120 PMC4166164

[37] Hermes DJ, Xu C, Poklis JL, et al. Neuroprotective effects of fatty acid amide hydrolase catabolic enzyme inhibition in a HIV-1 Tat model of neuroAIDS. Neuropharmacology 2018;141:55–65; doi: 10.1016/j.neuropharm.2018.08.013 30114402 PMC6296377

[38] Hermes DJ, Yadav-Samudrala BJ, Xu C, et al. GPR18 drives FAAH inhibition-induced neuroprotection against HIV-1 Tat-induced neurodegeneration. Exp Neurol 2021;341:113699; doi: 10.1016/j.expneurol.2021.113699 33736974 PMC8984429

[39] Grynkiewicz G, Poenie M, Tsien RY. A new generation of Ca^2+^ indicators with greatly improved fluorescence properties. J Biol Chem 1985;260(6):3440–3450. 3838314

[40] NIH National Library of Medicine. A Study to Evaluate a New Tablet Formulation of Lu AG06466 in Healthy Participants. Bethesda, MD. Available from: https://clinicaltrials.gov/study/NCT05028673?cond=AG06466&limit=50&rank=2#publications [Last accessed: October 27, 2023].

[41] Bruce-Keller AJ, Turchan-Cholewo J, Smart EJ, et al. Morphine causes rapid increases in glial activation and neuronal injury in the striatum of inducible HIV-1 Tat transgenic mice. Glia 2008;56(13):1414–1427; doi: 10.1002/glia.20708 18551626 PMC2725184

[42] Chauhan A, Turchan J, Pocernich C, et al. Intracellular human immunodeficiency virus Tat expression in astrocytes promotes astrocyte survival but induces potent neurotoxicity at distant sites via axonal transport. J Biol Chem 2003;278(15):13512–13519; doi: 10.1074/jbc.M209381200 12551932

[43] Hermes DJ, Jacobs IR, Key MC, et al. Escalating morphine dosing in HIV-1 Tat transgenic mice with sustained Tat exposure reveals an allostatic shift in neuroinflammatory regulation accompanied by increased neuroprotective non-endocannabinoid lipid signaling molecules and amino acids. J Neuroinflammation 2020;17(1):345; doi: 10.1186/s12974-020-01971-6 33208151 PMC7672881

[44] Yadav-Samudrala BJ, Gorman B, Dodson H, et al. Effects of acite D9-tetrahydrocannabinol on behavior and the endocannabinoid system in HIV-1 Tat transgenic female and male mice. Brain Res 2024;1822:148638; doi: 10.1016/j.brainres.2023.148638 37858856 PMC10873064

[45] Dempsey SK, Gesseck AM, Ahmad A, et al. Formation of HETE-EAs and dihydroxy derivatives in mouse kidney tissue and analysis by high-performance liquid chromatography tandem mass spectrometry. J Chromatogr B Analyt Technol Biomed Life Sci 2019;1126–1127:121748; doi: 10.1016/j.jchromb.2019.121748 PMC6935345 31437772

[46] Jacobs IR, Xu C, Hermes DJ, et al. Inhibitory control deficits associated with upregulation of CB(1)R in the HIV-1 Tat transgenic mouse model of hand. J Neuroimmune Pharmacol 2019;14(4):661–678; doi: 10.1007/s11481-019-09867-w 31372820 PMC6898753

[47] Xu C, Hermes DJ, Nwanguma B, et al. Endocannabinoids exert CB1 receptor-mediated neuroprotective effects in models of neuronal damage induced by HIV-1 Tat protein. Mol Cell Neurosci 2017;83:92–102; doi: 10.1016/j.mcn.2017.07.003 28733129 PMC5587778

[48] Fontana G, Valenti L, Raiteri M. Gp120 can revert antagonism at the glycine site of NMDA receptors mediating GABA release from cultured hippocampal neurons. J Neurosci Res 1997;49(6):732–738; doi: 10.1002/(SICI)1097-4547(19970915)49:6<732::AID-JNR7>3.0.CO;2-8 9335260

[49] Haughey NJ, Holden CP, Nath A, et al. Involvement of inositol 1,4,5-trisphosphate-regulated stores of intracellular calcium in calcium dysregulation and neuron cell death caused by HIV-1 protein tat. J Neurochem 1999;73(4):1363–1374; doi: 10.1046/j.1471-4159.1999.0731363.x 10501179

[50] Magnuson DS, Knudsen BE, Geiger JD, et al. Human immunodeficiency virus type 1 tat activates non-N-methyl-D-aspartate excitatory amino acid receptors and causes neurotoxicity. Ann Neurol 1995;37(3):373–380; doi: 10.1002/ana.410370314 7695237

[51] Liu Y, Jones M, Hingtgen CM, et al. Uptake of HIV-1 tat protein mediated by low-density lipoprotein receptor-related protein disrupts the neuronal metabolic balance of the receptor ligands. Nat Med 2000;6(12):1380–1387; doi: 10.1038/82199 11100124

[52] Esposito G, Ligresti A, Izzo AA, et al. The endocannabinoid system protects rat glioma cells against HIV-1 Tat protein-induced cytotoxicity. Mechanism and regulation. J Biol Chem 2002;277(52):50348–50354; doi: 10.1074/jbc.M207170200 12388547

[53] Mattson MP, Haughey NJ, Nath A. Cell death in HIV dementia. Cell Death Differ. 2005;12(Suppl 1):893–904; doi: 10.1038/sj.cdd.4401577 15761472

[54] Shin AH, Kim HJ, Thayer SA. Subtype selective NMDA receptor antagonists induce recovery of synapses lost following exposure to HIV-1 Tat. Br J Pharmacol 2012;166(3):1002–1017; doi: 10.1111/j.1476-5381.2011.01805.x 22142193 PMC3417425

[55] Buczynski MW, Parsons LH. Quantification of brain endocannabinoid levels: Methods, interpretations and pitfalls. Br J Pharmacol 2010;160(3):423–442; doi: 10.1111/j.1476-5381.2010.00787.x 20590555 PMC2931546

[56] Gonsiorek W, Lunn C, Fan X, et al. Endocannabinoid 2-arachidonyl glycerol is a full agonist through human type 2 cannabinoid receptor: Antagonism by anandamide. Mol Pharmacol 2000;57(5):1045–1050. 10779390

[57] Sugiura T, Kodaka T, Nakane S, et al. Evidence that the cannabinoid CB1 receptor is a 2-arachidonoylglycerol receptor. Structure-activity relationship of 2-arachidonoylglycerol, ether-linked analogues, and related compounds. J Biol Chem 1999;274(5):2794–2801; doi: 10.1074/jbc.274.5.2794 9915812

[58] Sugiura T, Kondo S, Kishimoto S, et al. Evidence that 2-arachidonoylglycerol but not N-palmitoylethanolamine or anandamide is the physiological ligand for the cannabinoid CB2 receptor. Comparison of the agonistic activities of various cannabinoid receptor ligands in HL-60 cells. J Biol Chem 2000;275(1):605–612; doi: 10.1074/jbc.275.1.605 10617657

[59] Di Marzo V, Stella N, Zimmer A. Endocannabinoid signalling and the deteriorating brain. Nat Rev Neurosci 2015;16(1):30–42; doi: 10.1038/nrn3876 25524120 PMC4471876

[60] Wodarski R, Bagdas D, Paris JJ, et al. Reduced intraepidermal nerve fibre density, glial activation, and sensory changes in HIV type-1 Tat-expressing female mice: Involvement of Tat during early stages of HIV-associated painful sensory neuropathy. Pain Rep 2018;3(3):e654; doi: 10.1097/PR9.0000000000000654 29922746 PMC5999412

[61] Cirino TJ, Alleyne AR, Duarte V, et al. Expression of human immunodeficiency virus transactivator of transcription (HIV-Tat(1–86)) protein alters nociceptive processing that is sensitive to anti-oxidant and anti-inflammatory interventions. J Neuroimmune Pharmacol 2022;17(1–2):152–164; doi: 10.1007/s11481-021-09985-4 33619645 PMC8380260

[62] Chi X, Amet T, Byrd D, et al. Direct effects of HIV-1 Tat on excitability and survival of primary dorsal root ganglion neurons: Possible contribution to HIV-1-associated pain. PLoS One 2011;6(9):e24412; doi: 10.1371/journal.pone.0024412 21912693 PMC3166319

[63] Fitting S, Scoggins KL, Xu R, et al. Morphine efficacy is altered in conditional HIV-1 Tat transgenic mice. Eur J Pharmacol 2012;689(1–3):96–103; doi: 10.1016/j.ejphar.2012.05.029 22659585 PMC3402587

[64] Toma W, Paris JJ, Warncke UO, et al. Persistent sensory changes and sex differences in transgenic mice conditionally expressing HIV-1 Tat regulatory protein. Exp Neurol 2022;358:114226; doi: 10.1016/j.expneurol.2022.114226 36096180 PMC10053560

[65] Crowe MS, Wilson CD, Leishman E, et al. The monoacylglycerol lipase inhibitor KML29 with gabapentin synergistically produces analgesia in mice. Br J Pharmacol 2017;174(23):4523–4539; doi: 10.1111/bph.14055 28963716 PMC5715597

[66] Kamimura R, Hossain MZ, Unno S, et al. Inhibition of 2-arachydonoylgycerol degradation attenuates orofacial neuropathic pain in trigeminal nerve-injured mice. J Oral Sci 2018;60(1):37–44; doi: 10.2334/josnusd.17-0005 29503395

[67] Kinsey SG, Long JZ, O'Neal ST, et al. Blockade of endocannabinoid-degrading enzymes attenuates neuropathic pain. J Pharmacol Exp Ther 2009;330(3):902–910; doi: 10.1124/jpet.109.155465 19502530 PMC2729802

[68] Kinsey SG, Naidu PS, Cravatt BF, et al. Fatty acid amide hydrolase blockade attenuates the development of collagen-induced arthritis and related thermal hyperalgesia in mice. Pharmacol Biochem Behav 2011;99(4):718–725; doi: 10.1016/j.pbb.2011.06.022 21740924 PMC3164582

[69] Wilkerson JL, Niphakis MJ, Grim TW, et al. The selective monoacylglycerol lipase inhibitor MJN110 produces opioid-sparing effects in a mouse neuropathic pain model. J Pharmacol Exp Ther 2016;357(1):145–156; doi: 10.1124/jpet.115.229971 26791602 PMC4809319

[70] Kinsey SG, Wise LE, Ramesh D, et al. Repeated low-dose administration of the monoacylglycerol lipase inhibitor JZL184 retains cannabinoid receptor type 1-mediated antinociceptive and gastroprotective effects. J Pharmacol Exp Ther 2013;345(3):492–501; doi: 10.1124/jpet.112.201426 23412396 PMC3657109

[71] Ignatowska-Jankowska B, Wilkerson JL, Mustafa M, et al. Selective monoacylglycerol lipase inhibitors: Antinociceptive versus cannabimimetic effects in mice. J Pharmacol Exp Ther 2015;353(2):424–432; doi: 10.1124/jpet.114.222315 25762694 PMC4407719

[72] Comelli F, Giagnoni G, Bettoni I, et al. The inhibition of monoacylglycerol lipase by URB602 showed an anti-inflammatory and anti-nociceptive effect in a murine model of acute inflammation. Br J Pharmacol 2007;152(5):787–794; doi: 10.1038/sj.bjp.0707425 17700715 PMC2190015

[73] Guindon J, Lai Y, Takacs SM, et al. Alterations in endocannabinoid tone following chemotherapy-induced peripheral neuropathy: Effects of endocannabinoid deactivation inhibitors targeting fatty-acid amide hydrolase and monoacylglycerol lipase in comparison to reference analgesics following cisplatin treatment. Pharmacol Res 2013;67(1):94–109; doi: 10.1016/j.phrs.2012.10.013 23127915 PMC3525790

[74] Woodhams SG, Wong A, Barrett DA, et al. Spinal administration of the monoacylglycerol lipase inhibitor JZL184 produces robust inhibitory effects on nociceptive processing and the development of central sensitization in the rat. Br J Pharmacol 2012;167(8):1609–1619; doi: 10.1111/j.1476-5381.2012.02179.x 22924700 PMC3525864

[75] Terrone G, Pauletti A, Salamone A, et al. Inhibition of monoacylglycerol lipase terminates diazepam-resistant status epilepticus in mice and its effects are potentiated by a ketogenic diet. Epilepsia 2018;59(1):79–91; doi: 10.1111/epi.13950 29171003

[76] Schlosburg JE, Blankman JL, Long JZ, et al. Chronic monoacylglycerol lipase blockade causes functional antagonism of the endocannabinoid system. Nat Neurosci 2010;13(9):1113–1119; doi: 10.1038/nn.2616 20729846 PMC2928870

[77] Chanda PK, Gao Y, Mark L, et al. Monoacylglycerol lipase activity is a critical modulator of the tone and integrity of the endocannabinoid system. Mol Pharmacol 2010;78(6):996–1003; doi: 10.1124/mol.110.068304 20855465

[78] Robinson-Papp J, Gensler G, Navis A, et al. Characteristics of motor dysfunction in longstanding human immunodeficiency virus. Clin Infect Dis 2020;71(6):1532–1538; doi: 10.1093/cid/ciz986 31587032 PMC7486845

[79] Kronemer SI, Mandel JA, Sacktor NC, et al. Impairments of motor function while multitasking in HIV. Front Hum Neurosci 2017;11:212; doi: 10.3389/fnhum.2017.00212 28503143 PMC5408028

[80] Hahn YK, Podhaizer EM, Farris SP, et al. Effects of chronic HIV-1 Tat exposure in the CNS: Heightened vulnerability of males versus females to changes in cell numbers, synaptic integrity, and behavior. Brain Struct Funct 2015;220(2):605–623; doi: 10.1007/s00429-013-0676-6 24352707 PMC4341022

[81] Joshi CR, Stacy S, Sumien N, et al. Astrocyte HIV-1 Tat differentially modulates behavior and brain MMP/TIMP balance during short and prolonged induction in transgenic mice. Front Neurol 2020;11:593188; doi: 10.3389/fneur.2020.593188 33384653 PMC7769877

[82] June HL, Tzeng Yang AR, Bryant JL, et al. Vitamin A deficiency and behavioral and motor deficits in the human immunodeficiency virus type 1 transgenic rat. J Neurovirol 2009;15(5–6):380–389; doi: 10.3109/13550280903350200 19995129 PMC3005340

[83] Moran LM, Booze RM, Webb KM, et al. Neurobehavioral alterations in HIV-1 transgenic rats: Evidence for dopaminergic dysfunction. Exp Neurol 2013;239:139–147; doi: 10.1016/j.expneurol.2012.10.008 23063600 PMC3698876

[84] Kesby JP, Najera JA, Romoli B, et al. HIV-1 TAT protein enhances sensitization to methamphetamine by affecting dopaminergic function. Brain Behav Immun 2017;65:210–221; doi: 10.1016/j.bbi.2017.05.004 28495611 PMC5537017

[85] Zhao X, Fan Y, Vann PH, et al. Long-term HIV-1 Tat expression in the brain led to neurobehavioral, pathological, and epigenetic changes reminiscent of accelerated aging. Aging Dis 2020;11(1):93–107; doi: 10.14336/AD.2019.0323 32010484 PMC6961778

[86] Bedse G, Bluett RJ, Patrick TA, et al. Therapeutic endocannabinoid augmentation for mood and anxiety disorders: Comparative profiling of FAAH, MAGL and dual inhibitors. Transl Psychiatry 2018;8(1):92; doi: 10.1038/s41398-018-0141-7 29695817 PMC5917016

[87] Aliczki M, Zelena D, Mikics E, et al. Monoacylglycerol lipase inhibition-induced changes in plasma corticosterone levels, anxiety and locomotor activity in male CD1 mice. Horm Behav 2013;63(5):752–758; doi: 10.1016/j.yhbeh.2013.03.017 23578952

[88] Aliczki M, Balogh Z, Tulogdi A, et al. The temporal dynamics of the effects of monoacylglycerol lipase blockade on locomotion, anxiety, and body temperature. Behav Pharmacol 2012;23(4):348–357; doi: 10.1097/FBP.0b013e3283564dfa 22750842

[89] Cosenza-Nashat MA, Bauman A, Zhao ML, et al. Cannabinoid receptor expression in HIV encephalitis and HIV-associated neuropathologic comorbidities. Neuropathol Appl Neurobiol 2011;37(5):464–483; doi: 10.1111/j.1365-2990.2011.01177.x 21450051 PMC3135748

[90] Swinton MK, Sundermann EE, Pedersen L, et al. Alterations in brain cannabinoid receptor levels are associated with HIV-associated neurocognitive disorders in the ART era: Implications for therapeutic strategies targeting the endocannabinoid system. Viruses. 2021;13(9):1742; doi: 10.3390/v13091742 34578323 PMC8473156

[91] Bosetti F. Arachidonic acid metabolism in brain physiology and pathology: Lessons from genetically altered mouse models. J Neurochem 2007;102(3):577–586; doi: 10.1111/j.1471-4159.2007.04558.x 17403135 PMC2084377

[92] Tallima H, El Ridi R. Arachidonic acid: Physiological roles and potential health benefits—A review. J Adv Res 2018;11:33–41; doi: 10.1016/j.jare.2017.11.004 30034874 PMC6052655

[93] Wang B, Wu L, Chen J, et al. Metabolism pathways of arachidonic acids: Mechanisms and potential therapeutic targets. Signal Transduct Target Ther 2021;6(1):94; doi: 10.1038/s41392-020-00443-w 33637672 PMC7910446

[94] Jadhav S, Nema V. HIV-associated neurotoxicity: The interplay of host and viral proteins. Mediators Inflamm 2021;2021:1267041; doi: 10.1155/2021/1267041 34483726 PMC8410439

[95] Yuan NY, Maung R, Xu Z, et al. Arachidonic acid cascade and eicosanoid production are elevated while LTC4 synthase modulates the lipidomics profile in the brain of the HIVgp120-transgenic mouse model of neuroHIV. Cells 2022;11(13):2123; doi: 10.3390/cells11132123 35805207 PMC9265961

[96] Wiebelhaus JM, Grim TW, Owens RA, et al. Delta9-tetrahydrocannabinol and endocannabinoid degradative enzyme inhibitors attenuate intracranial self-stimulation in mice. J Pharmacol Exp Ther 2015;352(2):195–207; doi: 10.1124/jpet.114.218677 25398241 PMC4293433

[97] Blankman JL, Simon GM, Cravatt BF. A comprehensive profile of brain enzymes that hydrolyze the endocannabinoid 2-arachidonoylglycerol. Chem Biol 2007;14(12):1347–1356; doi: 10.1016/j.chembiol.2007.11.006 18096503 PMC2692834

[98] Di Marzo V. Endocannabinoids: Synthesis and degradation. Rev Physiol Biochem Pharmacol 2008;160:1–24; doi: 10.1007/112_0505 18481028

[99] Sonnweber T, Pizzini A, Nairz M, et al. Arachidonic acid metabolites in cardiovascular and metabolic diseases. Int J Mol Sci 2018;19(11):3285; doi: 10.3390/ijms19113285 30360467 PMC6274989

[100] Benito C, Kim WK, Chavarria I, et al. A glial endogenous cannabinoid system is upregulated in the brains of macaques with simian immunodeficiency virus-induced encephalitis. J Neurosci 2005;25(10):2530–2536; doi: 10.1523/Jneurosci.3923-04.2005 15758162 PMC6725174

[101] Gorantla S, Makarov E, Roy D, et al. Immunoregulation of a CB2 receptor agonist in a murine model of neuroAIDS. J Neuroimmune Pharmacol 2010;5(3):456–468; doi: 10.1007/s11481-010-9225-8 20549374 PMC3109320

[102] Ramirez SH, Reichenbach NL, Fan S, et al. Attenuation of HIV-1 replication in macrophages by cannabinoid receptor 2 agonists. J Leukoc Biol 2013;93(5):801–810; doi: 10.1189/jlb.1012523 23463725 PMC3629438

[103] Ashton JC, Glass M. The cannabinoid CB2 receptor as a target for inflammation-dependent neurodegeneration. Curr Neuropharmacol 2007;5(2):73–80; doi: 10.2174/157015907780866884 18615177 PMC2435344

[104] Javed H, Azimullah S, Haque ME, et al. Cannabinoid type 2 (CB2) receptors activation protects against oxidative stress and neuroinflammation associated dopaminergic neurodegeneration in rotenone model of Parkinson's disease. Front Neurosci 2016;10:321; doi: 10.3389/fnins.2016.00321 27531971 PMC4969295

[105] Palazuelos J, Aguado T, Pazos MR, et al. Microglial CB2 cannabinoid receptors are neuroprotective in Huntington's disease excitotoxicity. Brain 2009;132(Pt 11):3152–3164; doi: 10.1093/brain/awp239 19805493

[106] Duarte EAC, Benevides ML, Martins ALP, et al. Female sex is strongly associated with cognitive impairment in HIV infection. Neurol Sci 2021;42(5):1853–1860; doi: 10.1007/s10072-020-04705-x 32929628

[107] Maki PM, Martin-Thormeyer E. HIV, cognition and women. Neuropsychol Rev 2009;19(2):204–214; doi: 10.1007/s11065-009-9093-2 19430907 PMC3716452

[108] Maki PM, Rubin LH, Springer G, et al. Differences in cognitive function between women and men with HIV. J Acquir Immune Defic Syndr 2018;79(1):101–107; doi: 10.1097/QAI.0000000000001764 29847476 PMC6092201

[109] Rubin LH, Neigh GN, Sundermann EE, et al. Sex differences in neurocognitive function in adults with HIV: Patterns, predictors, and mechanisms. Curr Psychiatry Rep 2019;21(10):94; doi: 10.1007/s11920-019-1089-x 31522330 PMC7673651

[110] Rubin LH, Sundermann EE, Dastgheyb R, et al. Sex differences in the patterns and predictors of cognitive function in HIV. Front Neurol 2020;11:551921; doi: 10.3389/fneur.2020.551921 33329301 PMC7732436

[111] Sundermann EE, Heaton RK, Pasipanodya E, et al. Sex differences in HIV-associated cognitive impairment. AIDS 2018;32(18):2719–2726; doi: 10.1097/QAD.0000000000002012 30407251 PMC6396274

[112] Paldino E, Balducci C, La Vitola P, et al. Neuroprotective effects of doxycycline in the R6/2 mouse model of Huntington's disease. Mol Neurobiol 2020;57(4):1889–1903; doi: 10.1007/s12035-019-01847-8 31879858 PMC7118056

[113] Santa-Cecilia FV, Leite CA, Del-Bel E, et al. The neuroprotective effect of doxycycline on neurodegenerative diseases. Neurotox Res 2019;35(4):981–986; doi: 10.1007/s12640-019-00015-z 30798507

