# Supplementary material for: Acute Effects of Monoacylglycerol Lipase Inhibitor ABX1431 on Neuronal Hyperexcitability, Nociception, Locomotion, and the Endocannabinoid System in HIV-1 Tat Male Mice
Source: Cannabis Cannabinoid Res. 2024 Dec 2;9(6):1500–13. doi: 10.1089/can.2023.0247 (PMC11685295; doi:10.1089/can.2023.0247)
Supplement: Supplementary Figure S1 [file can.2023.0247_suppl_figs1.docx]

**Supplemental Figure S1: Western Blot – Raw Images**


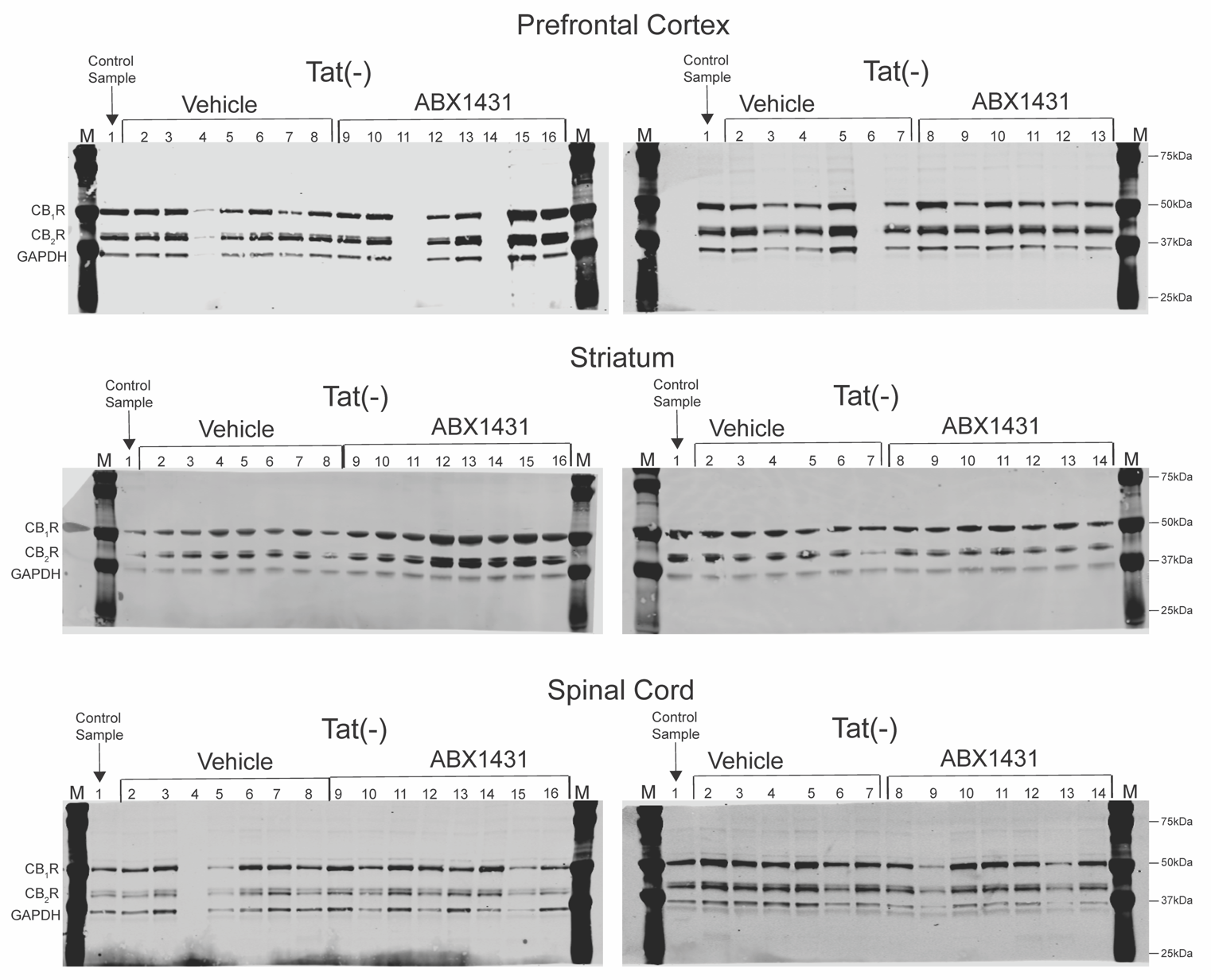


**A.**

**B.**

**C.**

**Figure S1. Original and unedited blots of CB_1_R and CB_2_R expression levels in the prefrontal cortex (A), striatum (B), and spinal cord (C).** Images show original CB_1_R, CB_2_R, and GAPDH Western blot gels for and male Tat transgenic mice used for **Figure 5**. For all Western blot gels lane 1 is a control sample represented on all blots. Male Tat(-) mice treated with vehicle (*n* = 7) are represented by lanes 2-8 and Tat(+) mice treated with vehicle (*n* = 6) are represented by lanes 2-7. Male Tat(-) mice treated with acute ABX1431 (*n* = 8) are represented by lanes 9-16 and male Tat(+) mice treated with acute ABX1431 (*n* = 7) are represented by lanes 8-14. Two samples for vehicle treated PFC Tat(-), one sample for vehicle treated PFC Tat(+), and one sample for vehicle treated SC Tat(-) were ost during snap freezing process. **(**M: molecular weights of marker protein (kDa).
