# Supplementary material for: Acute Effects of Monoacylglycerol Lipase Inhibitor ABX1431 on Neuronal Hyperexcitability, Nociception, Locomotion, and the Endocannabinoid System in HIV-1 Tat Male Mice
Source: Cannabis Cannabinoid Res. 2024 Dec 2;9(6):1500–13. doi: 10.1089/can.2023.0247 (PMC11685295; doi:10.1089/can.2023.0247)
Supplement: Supplementary Table S1 [file can.2023.0247_suppl_tables1.docx]

**Supplemental Table S1: Effects of acute ABX1431 (4mg/kg) on AEA, 2-AG, PEA, OEA, and AA levels (nmol/g) in four CNS regions.***

| **CNS Region** | **Lipids (nm/mg)** | **Genotype** | **Vehicle treatment**  **mean ± SEM** | **ABX1431 treatment**  **mean ± SEM** | **Treatment effect**  ***p*** | **Genotype**  **effect**  ***p*** | **Treatment x Genotype**  ***p*** |
| --- | --- | --- | --- | --- | --- | --- | --- |
| PFC | AEA | Tat (-) | 0.262 ± 0.035 | 0.339 ± 0.052 | 0.341 | 0.222 | 0.399 |
|  |  | Tat (+) | 0.356 ± 0.027 | 0.356 ± 0.027 |  |  |  |
|  | 2-AG | Tat (-) | 2.620 ± 0.791 | 4.517 ± 1.252 | 0.080 | 0.488 | 0.944 |
|  |  | Tat (+) | 1.787 ± 0.444 | 3.836 ± 1.083 |  |  |  |
|  | PEA | Tat (-) | 1.106 ± 0.130 | 1.114 ± 0.166 | 0.582 | 0.665 | 0.630 |
|  |  | Tat (+) | 0.987 ± 0.088 | 1.121 ± 0.035 |  |  |  |
|  | OEA | Tat (-) | 1.002 ± 0.102 | 1.071 ± 0.144 | 0.598 | 0.776 | 0.939 |
|  |  | Tat (+) | 1.043 ± 0.100 | 1.095 ± 0.046 |  |  |  |
|  | AA | Tat (-) | 471.16 ± 29.118 | 588.03 ± 99.00 | 0.194 | 0.562 | 0.818 |
|  |  | Tat (+) | 532.18 ± 43.581 | 614.52 ± 60.47 |  |  |  |
|  | | | | | | | |
| Str | AEA | Tat (-) | 0.185 ± 0.017 | 0.174 ± 0.013 | 0.701 | 0.171 | 0.684 |
|  |  | Tat (+) | 0.199 ± 0.014 | 0.199 ± 0.009 |  |  |  |
|  | 2-AG | Tat (-) | 5.392 ± 1.901 | 11.870 ± 2.932 | **0.011** | 0.270 | 0.778 |
|  |  | Tat (+) | 3.602 ± 0.618 | 8.874 ± 1.993 |  |  |  |
|  | PEA | Tat (-) | 2.345 ± 0.289 | 1.942 ± 0.085 | 0.599 | 0.068 | 0.081 |
|  |  | Tat (+) | 1.706 ± 0.083 | 1.927 ± 0.108 |  |  |  |
|  | OEA | Tat (-) | 1.887 ± 0.169 | 1.624 ± 0.058 | 0.523 | 0.336 | 0.090 |
|  |  | Tat (+) | 1.587 ± 0.069 | 1.710 ± 0.089 |  |  |  |
|  | AA | Tat (-) | 543.44 ± 56.67 | 673.55 ± 33.048 | 0.299 | **0.005** | **0.053** |
|  |  | Tat (+) | 756.53 ± 41.93 | 715.80 ± 28.371 |  |  |  |
|  | | | | | | | |
| Crb | AEA | Tat (-) | 0.130 ± 0.012 | 0.134 ± 0.016 | 0.533 | 0.881 | 0.717 |
|  |  | Tat (+) | 0.127 ± 0.013 | 0.142 ± 0.015 |  |  |  |
|  | 2-AG | Tat (-) | 8.635 ± 0.866 | 24.79 ± 4.402 | **<0.001** | 0.671 | 0.707 |
|  |  | Tat (+) | 8.490 ± 1.153 | 22.414 ± 2.563 |  |  |  |
|  | PEA | Tat (-) | 1.148 ± 0.085 | 1.334 ± 0.169 | 0.322 | 0.122 | 0.604 |
|  |  | Tat (+) | 1.018 ± 0.065 | 1.076 ± 0.095 |  |  |  |
|  | OEA | Tat (-) | 1.107 ± 0.085 | 1.219 ± 0.174 | 0.495 | 0.303 | 0.844 |
|  |  | Tat (+) | 1.000 ± 0.047 | 1.062 ± 0.112 |  |  |  |
|  | AA | Tat (-) | 795.73 ± 61.95 | 876.38 ± 140.91 | 0.408 | 0.767 | 0.932 |
|  |  | Tat (+) | 754.63 ± 69.33 | 853.59 ± 105.77 |  |  |  |
|  | | | | | | | |
| SC | AEA | Tat (-) | 0.041 ± 0.005 | 0.046 ± 0.008 | 0.791 | 0.304 | 0.808 |
|  |  | Tat (+) | 0.055 ± 0.010 | 0.055 ± 0.016 |  |  |  |
|  | 2-AG | Tat (-) | 16.978 ± 2.145 | 76.473 ± 22.755 | **0.013** | 0.907 | 0.984 |
|  |  | Tat (+) | 14.837 ± 2.417 | 73.412 ± 35.087 |  |  |  |
|  | PEA | Tat (-) | 1.518 ± 0.247 | 1.928 ± 0.483 | 0.376 | 0.819 | 0.976 |
|  |  | Tat (+) | 1.634 ± 0.214 | 2.016 ± 0.590 |  |  |  |
|  | OEA | Tat (-) | 1.048 ± 0.166 | 1.312 ± 0.302 | 0.426 | 0.657 | 0.932 |
|  |  | Tat (+) | 1.206 ± 0.188 | 1.419 ± 0.411 |  |  |  |
|  | AA | Tat (-) | 799.51 ± 111.14 | 989.67 ± 253.56 | 0.987 | 0.754 | 0.334 |
|  |  | Tat (+) | 930.76 ± 189.26 | 734.28 ± 171.48 |  |  |  |

*Levels of endocannabinoid ligands and related lipids the prefrontal cortex, striatum, cerebellum, and spinal cord of Tat(-) and Tat(+) male mice exposed to acute 4 mg/kg CBD or vehicle expressed as mean ± SEM in nmol/g. A two-way analysis of variance (ANOVA) for each lipid molecule was conducted with treatment and genotype as between-subjects factors. *p*-values are presented from ANOVA results. Bolded and red font values denote significant differences at *p* ≤ 0.05; mean ± SEM.
